# Mismatch Repair Protein Deficiency and Its Relationship with Clinicopathological Factors in Endometrial Cancer: A Retrospective Study

**DOI:** 10.7150/jca.112935

**Published:** 2025-06-12

**Authors:** Okan Aytekin, Nesibe Cesur, Sercan Gozel, Sezin Eda Karsli, Abdurrahman Alp Tokalioglu, Fatih Kilic, Zeliha Firat Cuylan, Ilker Selcuk, Gunsu Kimyon Comert, Fazli Erdogan, Taner Turan

**Affiliations:** 1 Department of Gynecologic Oncology, Ankara Bilkent City Hospital, Ankara, Turkey 06800.; 2 Department of Pathology, Ankara Bilkent City Hospital, Ankara, Turkey 06800.

**Keywords:** endometrial cancer, immunohistochemistry, mismatch repair protein-deficient

## Abstract

**Background:** The present study aimed to determine the frequency of mismatch repair (MMR) protein expression loss, as identified using immunohistochemistry (IHC), in tumor cells of endometrial cancer patients and the potential associations between this loss of expression and various clinicopathological characteristics.

**Methods:** The preparations were considered positive if tumor cells showed immunoreactivity that was equal to or stronger than that of positive controls and negative if tumor cells completely lost immunoreactivity. MMR proficiency was defined as positive IHC staining of all four proteins [MutL homolog 1 (MLH1), MutS homolog 2, MutS homolog 6 and PMS1 homolog 2 (PMS2)]. If at least one of them showed negative IHC staining, this was interpreted as mismatch repair protein deficiency (dMMR).

**Results:** A total of 154 patients who met the criteria were included in this study. dMMR was observed in 54 (35.1%) patients in the study group. The MLH1 and PMS2 proteins were the most frequently lost, observed in 44 (28.8%) and 43 (27.9%) patients, respectively. Patients with dMMR were significantly older. However, there were no observed associations between dMMR and other clinicopathological factors.

**Conclusions:** In conclusion, a notable association between the expression of MMR proteins and the age of the patient was observed in this cohort. No significant associations were detected between other clinical, surgical or pathological factors and MMR protein expression.

## Introduction

Endometrial cancer is the most common gynecologic malignancy, and its incidence continues to increase in both developed and developing countries 1, 2. A group of endometrial cancers are known to be associated with Lynch syndrome, an autosomal dominant disease caused by germline mutations in mismatch repair (MMR) genes 3. Patients with Lynch syndrome have a lifetime risk of endometrial and colon cancer of 40-60% 3, 4.

MMR gene mutations are considered to be important for the tumorigenesis of endometrial cancers 5. Among these cases, 80-90% are linked to sporadic disease, primarily resulting from hypermethylation of the MutL homolog 1 (MLH1) promoter 6, 7. The remaining 10-20% of mismatch repair protein deficiency (dMMR) cases are associated with hereditary Lynch syndrome, an autosomal dominant disorder caused by pathogenic germline mutations in MLH1, PMS2 homolog 2 (PMS2), MutS homolog 2 (MSH2) and/or MutS homolog 6 (MSH6) 8, 9. The cause of the dMMR status identified with immunohistochemistry (IHC) is revealed using MLH1 promoter methylation testing and germline mutation testing.

Despite the considerable amount of research conducted on the deficit of MMR in colorectal cancer, there has been comparatively less exploration of the role of MMR in endometrial cancer. Lynch syndrome, which accounts for 10-20% of dMMR cases, is associated with a significantly increased lifetime risk of endometrial and other cancers 8. Identifying Lynch syndrome is important for genetic counseling and treatment decisions, including immunotherapy. The identification of the MSI phenotype in endometrial cancer holds significant importance due to the high prevalence of these tumors.

In endometrial cancer, the relationship between tumors with a loss of MMR protein expression and survival outcomes has not yet been fully established. There have been reports indicating that patients with endometrial cancer whose tumors lack MMR protein expression have a substantially higher survival rate. However, some studies do not support this conclusion 10-15.

The present study aimed to determine the frequency of MMR protein expression loss, as identified using IHC, in tumor cells in patients with endometrial cancer and the potential associations between this loss of expression and various clinicopathological characteristics.

## Materials and Methods

The present study was a retrospective cohort study. Patients who were operated on at our clinic due to endometrial cancer between September 2019 and March 2023 were included in this study. All patients underwent total hysterectomy and bilateral salpingo-oophorectomy as part of the standard surgery. Systematic pelvic and/or paraaortic lymphadenectomy, omentectomy and tumor debulking were included in the surgical procedure according to the intraoperative frozen/section result. The patients were staged according to the International Federation of Gynecology and Obstetrics (FIGO) 2009 criteria 16. Patients who lacked information concerning the MMR protein expression status in the postoperative pathology results were excluded. The Ankara Bilkent City Hospital ethics committee evaluated and approved the research procedure (IRB: E2-23-4782, dated 23 August 2023) in accordance with the Declaration of Helsinki, and this manuscript conformed with the Enhancing the QUAlity and Transparency of Health Research (EQUATOR) network guidelines.

Fresh biopsy or surgical tissue samples were fixed with 10% neutral-buffer formalin for 8 to 24h. According to the requirements of pathological technical specifications, sampling, dehydration, and embedding into paraffin block. Formalin-fixed, paraffin-embedded (FFPE) endometrial cancer blocks, which had high-quality tissue morphology, were used to prepare 4 µm sections on positively charged glass slides. No other thicknesses had been validated. Slides should be stained immediately. As the antigenicity of cut tissue sections may diminish over time and may be compromised 45 days after cutting from the FFPE tissue block, the slides were stained immediately. Immunostaining was carried out on the Ventana Benchmark ultra-automated stainer (Roche Tissue Diagnostics; Roche Diagnostics, Ltd.). The VENTANA MMR RxDx Panel (Roche Tissue Diagnostics; Roche Diagnostics, Ltd.) includes VENTANA anti-MLH1 (M1) Mouse Monoclonal Primary Antibody, VENTANA anti-MSH2 (G219-1129) Mouse Monoclonal Primary Antibody, VENTANA anti-MSH6 (SP93) Rabbit Monoclonal Primary Antibody and VENTANA anti-PMS2 (A16-4) Mouse Monoclonal Primary Antibody.

These antibodies have been optimized for specific incubation times, but the user must validate results obtained with this reagent. The effect of varying time and temperature of the antigen retrieval (cell conditioning) and antibody incubation from the recommended staining may result in sub-optimal staining and false deficient and false proficient results. Any deviation from recommended test procedures may invalidate expected results. Appropriate controls must be employed and documented.

The tissue slices were deparaffinized, antigen retrieval was performed using a reaction buffer containing 0.3% carrier protein, and endogenous peroxidase was blocked using a pre-primary peroxidase inhibitor. Subsequently, tissue slices were incubated with primary antibodies at 36 ˚C (MLH1, 24 min; MSH2 and MSH6, 12 min; PMS2, 36 min). Specific antigen/antibody reactions were visualized with the OptiView DAB IHC Detection Kit for PMS2 and the ultraView Universal DAB IHC Detection Kit for MLH1, MSH2 and MSH6. Finally, counterstaining was performed using hematoxylin, and post-counterstaining was performed by bluing for 4 min.

**Figure 1:**
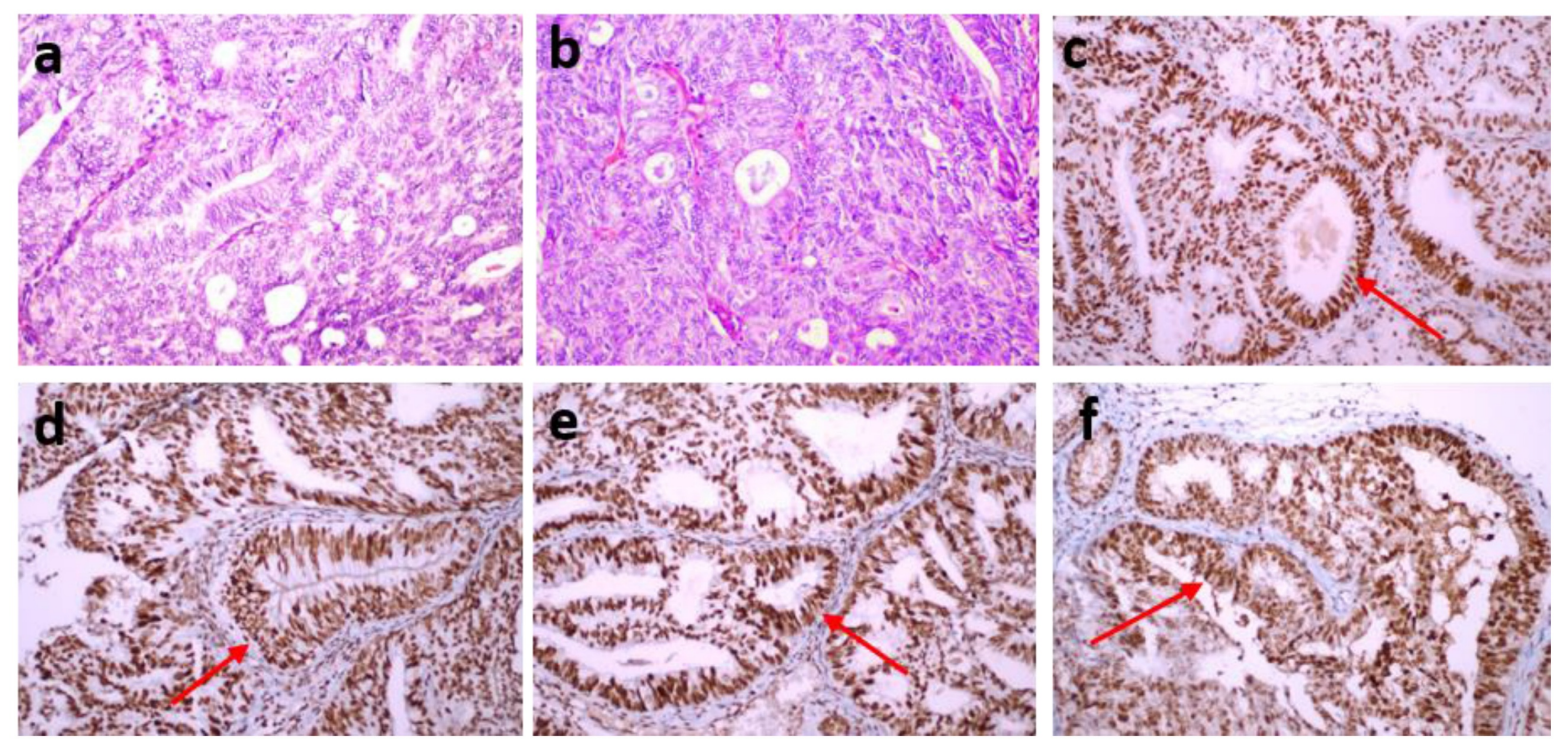
(a,b) An endometrioid endometrial cancer case (FIGO Grade 2) (hematoxylin-eosin stain x200). All four proteins [MLH1 (c), MSH2 (d), MSH6 (e) and PMS2 (f)] reflected positive IHC staining, MMR proficiency (pMMR) (IHC staining x200).

**Figure 2:**
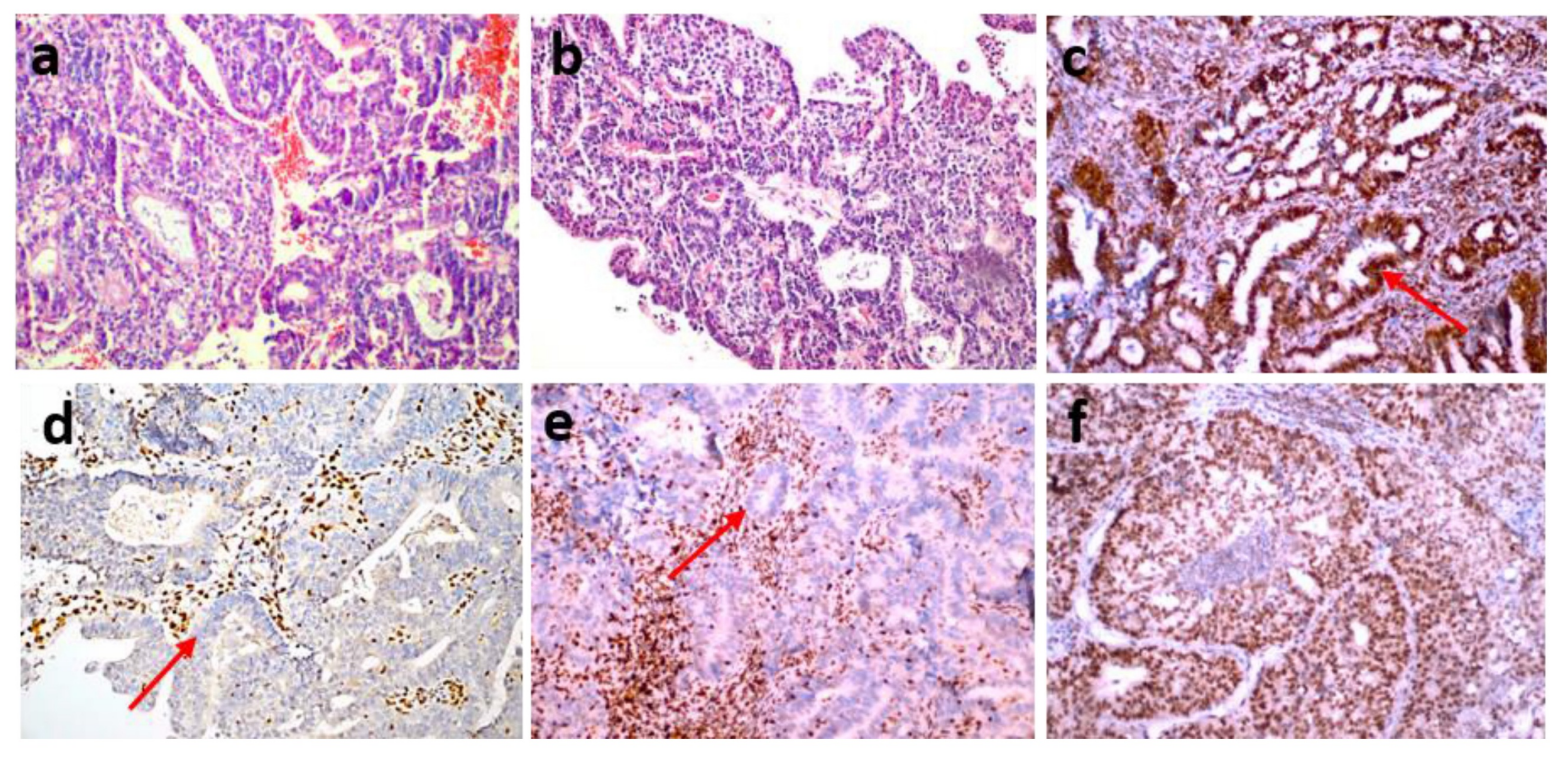
(a,b) An endometrioid endometrial cancer case (FIGO Grade 2) (hematoxylin-eosin stain x200) showing the loss of MSH2 (d) and MSH6 (e) expression, nuclear staining of MLH1 (c) and PMS2 (f) MMR deficiency (dMMR) (IHC staining x200).

Each of the stained preparations was examined by two specialist histopathologists. They detected staining in the nuclei of tumor epithelial cells compared with the positive staining of stromal cells and lymphocytes as positive internal controls and the positive staining of normal appendix epithelial cells and lymphocytes of the subepithelial area as positive external controls. The preparations were considered positive if tumor cells showed immunoreactivity that may be focal but equal to or stronger than that of positive controls and negative if tumor cells completely lost immunoreactivity. Mismatch repair protein proficiency (pMMR) was defined as positive IHC staining of all four proteins (MLH1, MSH2, MSH6 and PMS2) (Fig. 1). If at least one of them showed negative IHC staining, this was interpreted as dMMR (Fig. 2) 17. Punctate nuclear staining considered negative.

Version 22.0 of the Statistical Package for the Social Sciences (IBM Corp.) was used to conduct statistical analyses. Continuous variables were summarized as mean ± standard deviation or median (min-max) and analyzed using the ANOVA test. Categorical variables were summarized as numbers and percentages and analyzed using the χ² test. A p-value of <0.05 was considered statistically significant. A Venn diagram (R package) was generated with four sets (MLH1, MSH2, MSH6 and PMS2) to show their intersections 18.

## Results

A total of 154 patients who met the criteria were included in this study (Table 1). The median age of the patients was 63 years (range, 31-86 years). The median tumor size was 40 mm (range, 4-150 mm). The most common stage was FIGO IA, which was observed in 78 (50.6%) patients. The most common tumor type was the endometrioid type, which was observed in 141 (91.6%) patients. In 16 (10.4%) patients, the extent of myometrial invasion was classified as 'no invasion', whereas in 5 (3.2%) patients, serosal invasion was identified. Additionally, lymph node metastasis was observed in 18 (17.8%) patients, peritoneal cytology in 4 (2.6%) patients, adnexal metastasis in 7 (4.5%) patients, omental metastasis in 4 (2.6%) patients, parametrial involvement in 4 (2.6%) patients and lymphovascular space invasion in 43 (27.9%) patients (Table 1).

**Figure 3:**
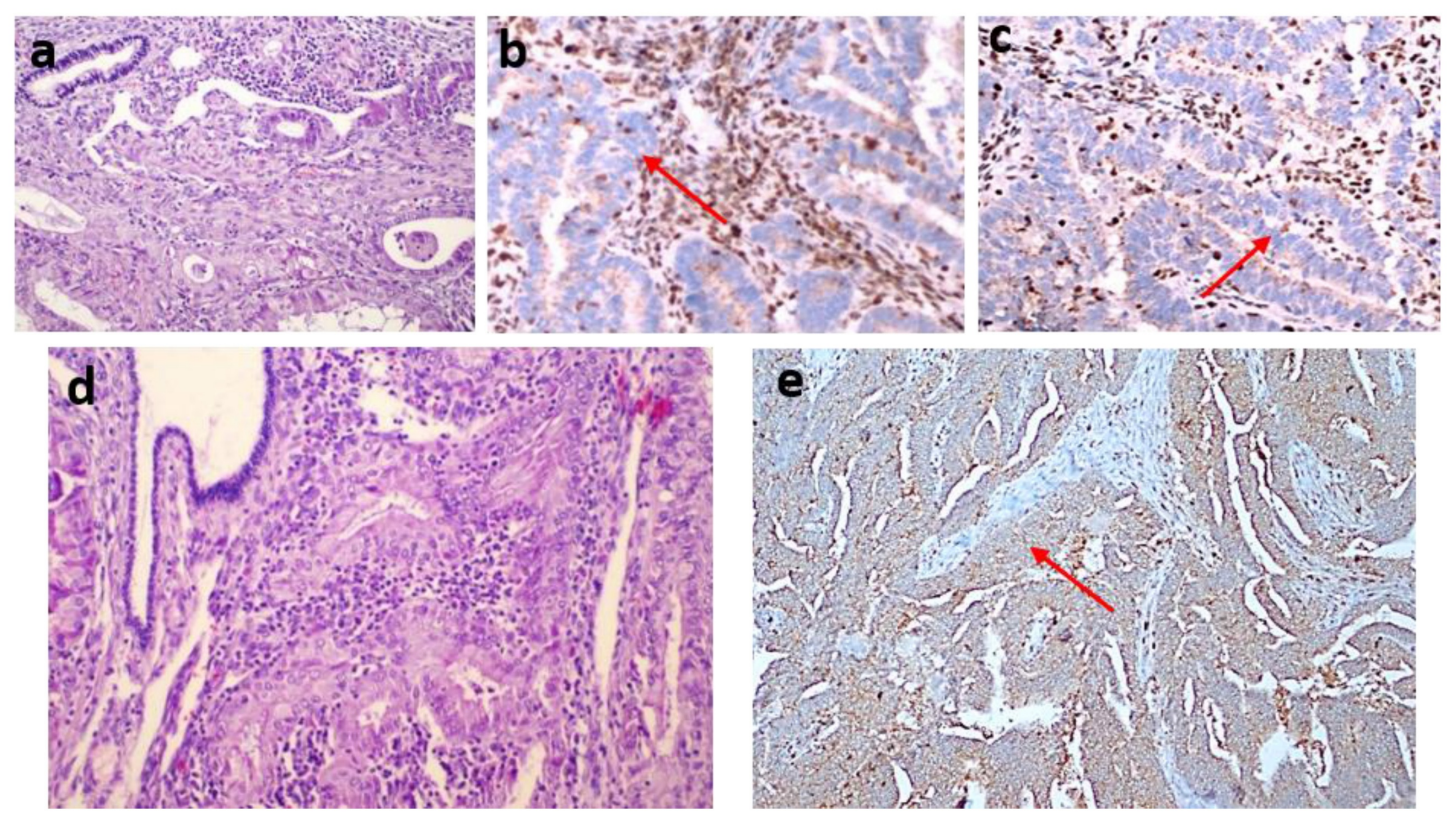
Two endometrioid carcinoma cases (a, d) (hematoxylin-eosin stain x200). The first one (a) shows the loss of MLH1 (b) and PMS2 (c) expression (IHC staining x200). The second one (d) shows the loss of PMS2 (e) (IHC staining x100).

**Figure 4:**
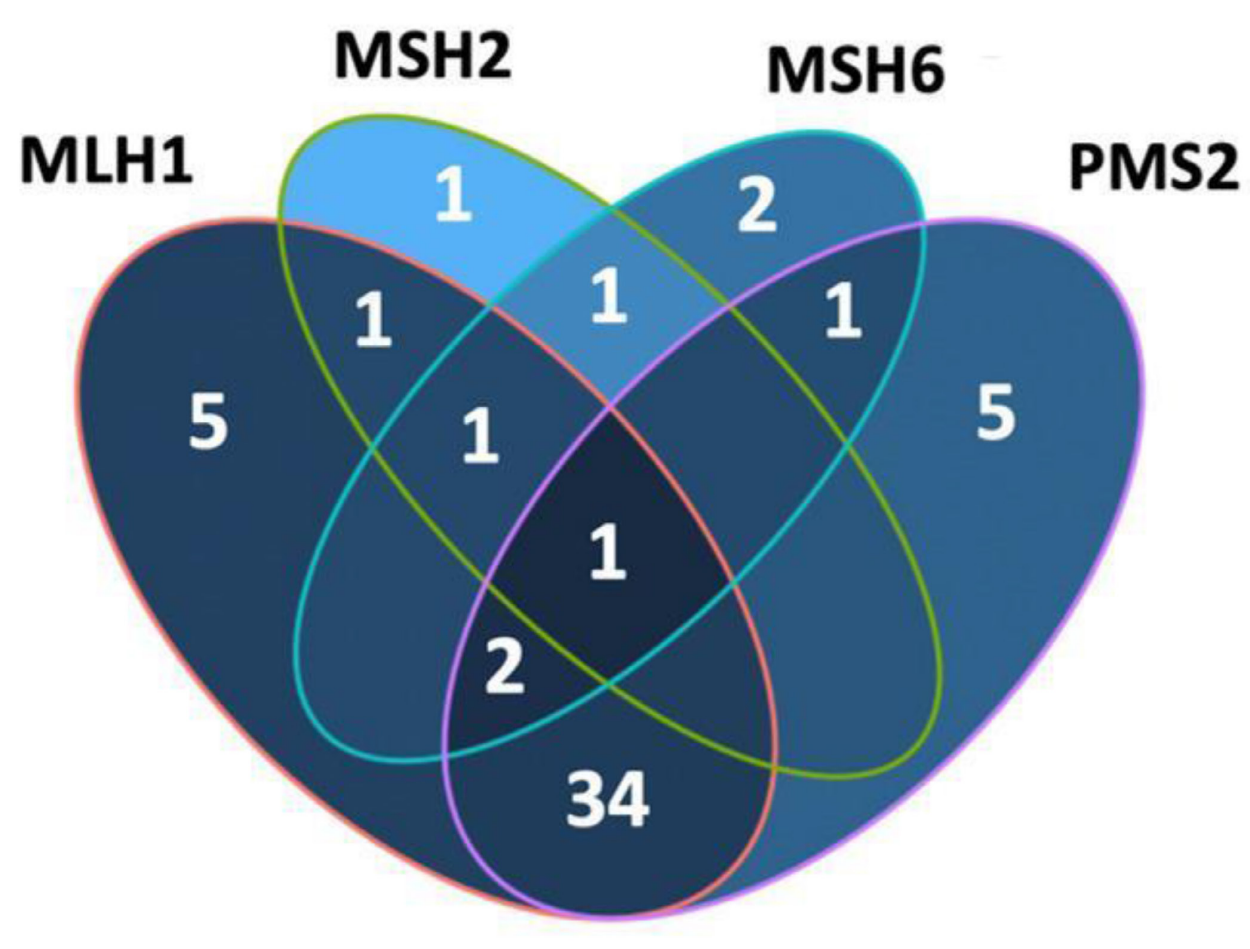
Venn diagram was created (in R package) with four sets (MLH1, MSH2, MSH6 and PMS2) to show their intersections.

dMMR was observed in 54 (35.1%) patients in the study group. The MLH1 and PMS2 proteins were the most frequently lost, observed in 44 (28.8%) and 43 (27.9%) patients, respectively. dMMR was observed in one protein in 13 (8.4%) patients, in two proteins in 37 (24%) patients, in three proteins in 3 (1.9%) patients and in four proteins in 1 (0.6%) patient (Table 2). The examination of the association between dMMR revealed that the most prevalent association observed was between MLH1 and PMS2. While isolated MLH1 and PMS2 losses were observed in 34 patients, isolated MLH1 loss was observed in 5 patients, and isolated PMS2 loss was observed in 5 patients (Fig. 3). Loss of MLH1, MSH2, MSH6 and PMS2 protein expression together was observed in only one patient (Fig. 4). In Figs. 1 and 2, proficiency and deficiency specimen images of MLH1, MSH2, MSH6 and PMS2 in IHC staining of cases with endometrioid endometrial cancer (FIGO grade 2) are presented.

The patient group with dMMR was statistically significantly older than the group with pMMR (*p*=0.022; Table 3). However, no associations were observed between dMMR and various other factors, such as tumor size, histopathology, FIGO 2009 stage, lymphadenectomy, presence of lymph node metastasis, lymphovascular space invasion, myometrial invasion degree, cervical involvement status, peritoneal cytology, omental metastasis status, parametrial spread status and adnexal metastasis.

## Discussion

The present study aimed to assess the prevalence of dMMR in tumor cells among patients diagnosed with endometrial cancer. The findings revealed that 35.1% of the patients exhibited dMMR in their tumor cells. The most frequently detected protein expression losses were in MLH1 and PMS2, with only one patient exhibiting loss of expression of all four proteins. The group with dMMR was older than the group with pMMR. However, other clinical, surgical and pathological factors were similar among the patient groups.

The primary role of the DNA MMR mechanism is to detect and correct errors that occur in DNA replication, ensuring the accuracy and integrity of the replication process 19. dMMR is characterized by the functional impairment of MLH1, PMS2, MSH2 and MSH6 proteins, leading to the dysfunction of the MMR system. This system is of critical significance in preserving genomic integrity. Microsatellites refer to short tandem repeats distributed throughout the genome. MSI refers to the alteration in the length of microsatellites caused by the insertion or deletion of repeat units during the process of DNA replication, which occurs due to the failure of the MMR system to rectify these errors. The primary cause of MSI is the absence of MMR protein expression, and thus, the identification of protein deletions can serve as an indirect indicator of MSI status 20-22.

In this study, loss of MLH1 and PMS2 was observed in 28.8% and 27.9% of patients, respectively. In a study by Backes *et al.* 23 examining the results of 140 patients with endometrial cancer, dMMR was observed in 21% of the patients, and the most frequently detected protein expression loss was MLH1 and PMS2, observed in 17.1% of the patients. In a study by Doghri *et al.* 24 on 44 patients, dMMR was detected in 22.7% of the patients, and the most frequently detected protein expression loss was MLH1 and PMS2, similar to the present study.

In this study, the coexistence of two dMMRs was detected in 68.5% of the patients, whereas expression loss of all four proteins was observed in only 1.8% of the patients. In a study by Wang *et al.* 25, the loss of two proteins was observed in 83.7% of the patients with loss of expression, while the loss of four proteins was not observed at all. In a study by Kato *et al.* 26, the loss of two proteins was observed in 47.3% of patients with loss of expression, while the loss of four proteins was observed in 2.6% of patients.

The relationship between dMMR and clinical, surgical and pathological factors is not clear. In the present study, only older age was found to be significantly more common in the group with dMMR. Similarly, a study by Wang *et al.* 25 examining the results of 333 patients with endometrial cancer demonstrated that the group with dMMR was markedly older. Unlike the present study, Kato *et al.* 26 showed that endometrioid cell type, low grade and early stage (FIGO stage I/II) were more common in the group with dMMR. The literature includes studies suggesting an association between dMMR and low-grade, early-stage disease 24, 27, as well as research indicating its link to high-grade, advanced-stage disease 28-30. In a study including 312 patients by de Freitas *et al.* 31, endometrioid cell type and lymphovascular space invasion positivity were more common in the dMMR group. In a study by Chaowiwatkun *et al.* 32 examining the results of 207 patients, the rate of deep myometrial invasion was found to be higher in the patient group with dMMR.

The molecular classification was established using data from The Cancer Genome Atlas and Proactive Molecular Risk Classifier for Endometrial Cancer, with a specific emphasis on molecular and IHC markers 33-35. The dMMR group constitutes a subgroup of the molecular classification of FIGO 2023 staging 34. Therefore, molecular markers are now being utilized as a prognostic factor. As the application of these prognostic factors has developed in clinical practice, the utilization of monoclonal antibodies in the treatment of patients with dMMR tumors has become important 36. Studies on the use of pembrolizumab, an anti-programmed death-1 monoclonal antibody, and lenvatinib, which inhibits vascular endothelial growth factor receptor 1, are ongoing 37, 38.

Lynch syndrome is an inherited disorder caused by germline mutations in mismatch repair (MMR) genes, which significantly increases the lifetime risk of developing endometrial cancer 39. Although this study focuses on the loss of MMR protein expression detected using immunohistochemistry, the potential contribution of germline mutations and hereditary conditions such as Lynch syndrome requires further exploration. To better identify patients with Lynch syndrome, comprehensive genetic counseling and molecular diagnostic testing should be integrated into the clinical management of individuals with MMR-deficient tumors.

Given the association between dMMR and Lynch syndrome, identifying patients with dMMR is crucial for further genetic evaluation. Patients with dMMR, particularly those with isolated MSH2 or MSH6 loss, may benefit from genetic counseling and germline testing to identify potential Lynch syndrome cases, which could impact both patient management and familial cancer risk assessment.

The main limitation of the present study pertains to its retrospective design. Another limitation is that, due to the limited number of patients with MSH2 and MSH6 protein deficiency, MMR proteins could not be compared among themselves. Due to the retrospective design of this study, data on MLH1 promoter hypermethylation were not available, which represents another limitation of our study. Future studies integrating hypermethylation testing are essential to better elucidate the etiology of MLH1-deficient tumors. This investigation has a large cohort for a single center, which is one of its notable advantages. Furthermore, only specialized gynecologic oncologists performed the surgical procedures and only specialized histopathologists conducted the subsequent pathological evaluations, which is an important strength of this study. Since the use of dMMR in clinical practice is novel, there are insufficient data on the survival outcomes of patients. This represents a limitation, and future studies incorporating survival data could offer further insights into the relationship between MMR deficiency and survival outcomes.

## Conclusions

IHC testing revealed that dMMR was present in 35% of endometrial cancer cases. The loss of expression of MLH1 and PMS2 was observed most frequently. A notable association was observed in the present cohort between the expression of MMR proteins and the age of the patient. No significant associations were detected between other clinical, surgical or pathological factors and MMR protein expression. The findings of the studies documented in the literature exhibit great variability, and thus, further studies are required.

## Figures and Tables

**Table 1:** General features (n:154 patients)

Characteristics		Mean±SD	Median (range)
Age, years		62.8±10.12	63 (31-86)
Tumor size (mm)		42.3±26.02	40 (4-150)
Totally removed lymph node count		44.5±21.4	43 (1-112)
Totally metastatic lymph node count		3.7±3.0	3 (1-12)
		n	%
FIGO 2009 stage	IA	78	50.6
	IB	43	27.9
	II	11	7.1
	IIIA	2	1.3
	IIIB	1	0.6
	IIIC1	6	3.9
	IIIC2	10	6.5
	IVA	-	-
	IVB	3	1.9
Histopathology	Endometrioid	141	91.6
	Serous	5	3.2
	Clear cell	1	0.6
	Mixed	7	4.5
Lymphadenectomy	Not performed	53	34.4
	Performed	101	65.6
Lymph node metastasis ^1^	No	83	82.2
	Yes	18	17.8
Metastatic lymph node site ^1^	Isolated pelvic	6	5.9
	Isolated paraaortic	4	3.9
	Pelvic and paraaortic	8	7.9
DMI	No invasion	16	10.4
	Invasion <1/2	67	43.5
	Invasion ≥1/2 ^2^	66	42.9
	Serosal invasion	5	3.2
Lymphovascular space invasion	Negative	111	72.1
	Positive	43	27.9
Cervical invasion	No invasion	132	85.7
	Glandular	4	2.6
	Stromal ± glandular	18	11.7
Peritoneal cytology	Negative	150	97.4
	Positive	4	2.6
Adnexal metastasis	Negative	147	95.5
	Positive	7	4.5
Omental metastasis	Negative	150	97.4
	Positive	4	2.6
Parametrial involvement	Negative	150	97.4
	Positive	4	2.6

^1^: The 101 patients performed lymphadenectomy was evaluated ^2^: Except serosal invasion **DMI:** Depth of myometrial invasion

**Table 2:** Mismatch Repair Protein Deficiency

Features		n	%
Mismatch repair protein deficiency	Negative	100	64.9
	Positive	54	35.1
MLH1 deficiency	Negative	106	68.8
	Positive	44	28.8
	Unidentified	4	2.6
MSH2 deficiency	Negative	149	96.8
	Positive	5	3.2
MSH6 deficiency	Negative	146	94.8
	Positive	8	5.2
PMS2 deficiency	Negative	105	68.2
	Positive	43	27.9
	Unidentified	6	3.9
Number of mismatch repair protein deficiency	0	100	64.9
	1	13	8.4
	2	37	24
	3	3	1.9
	4	1	0.6

**Table 3:** The Relationship with Mismatch Repair Protein Deficiency and Clinico-Pathologic Factors

Features		Mismatch Repair Protein Deficiency	
		Negative	Positive
		Median (range)	Median (range)
Age, years		61 (31-86)	67.5 (39-82)
*p* Value		*0.022*	
Tumor size, mm		40 (5-150)	40 (4-100)
*p* Value		*0.751*	
Preoperative CA125 level, IU/ml		14 (2-2781)	18 (4-17.880)
*p* Value		*0.214*	
		n (%)	n (%)
Histopathology	Endometrioid	93 (93)	48 (88.9)
	Non-endometrioid	7 (7)	6 (11.1)
*p* Value		*0.381*	
FIGO 2009 stage	I-II	84 (84)	48 (88.9)
	III-IV	16 (16)	6 (11.1)
*p* Value		*0.408*	
Lymphadenectomy	Not performed	33 (33)	20 (37)
	Performed	67 (67)	34 (63)
*p* Value		*0.615*	
Lymph node metastasis	Negative	55 (82.1)	28 (82.4)
	Positive	12 (17.9)	6 (17.6)
*p* Value		*0.974*	
DMI	No invasion or DMI <1/2	*55 (57.9)*	*28 (51.9)*
	DMI ≥1/2 ^1^	40 (42.1)	26 (48.1)
*p* Value		*0.475*	
Uterine serosal invasion	Negative	95 (95)	54 (100)
	Positive	5 (5)	0 (0)
*p* Value		*0.095*	
Lymphovascular space invasion	Negative	69 (69)	42 (77.8)
	Positive	31 (31)	12 (22.2)
*p* Value		*0.247*	
Uterine cervical invasion	Negative	85 (85)	47 (87)
	Positive ^2^	15 (15)	7 (13)
*p* Value		*0.730*	
Peritoneal cytology	Negative	96 (96)	54 (100)
	Positive	4 (4)	0 (0)
*p* Value		*0.136*	
Adnexal metastasis	Negative	94 (94)	53 (98.1)
	Positive	6 (6)	1 (1.9)
*p* Value		*0.238*	
Omental metastasis	Negative	96 (96)	54 (100)
	Positive	4 (4)	0 (0)
*p* Value		*0.136*	
Parametrial involvement	Negative	96 (96)	54 (100)
	Positive	4 (4)	0 (0)
*p* Value		*0.136*	
Extra-uterine corporal spread	Negative	74 (74)	44 (81.5)
	Positive	26 (26)	10 (18.5)
*p* Value		*0.295*	

**DMI:** Depth of myometrial invasion **^1^:** Except uterine serosal invasion (n=5) **^2^:** Stromal and/or glandular

## Abbreviations

EQUATOR: Enhancing the QUAlity and Transparency of health Research
FIGO: International Federation of Gynecology and Obstetrics
FFPE: Formalin-Fixed Paraffin-Embedded
IHC: Immunohistochemistry
MLH1: MutL Homolog 1
MSH2: MutS Homolog 2
MSH6: MutS Homolog 6
MSI: Microsatellite instability
MMR: Mismatch repair
dMMR: mismatch repair protein deficiency
pMMR: mismatch repair protein proficiency
PMS2: PMS1 Homolog 2
